# P254: The hospital-acquired infections in regional hospital in Niger Tahoua

**DOI:** 10.1186/2047-2994-2-S1-P254

**Published:** 2013-06-20

**Authors:** H Djibo, M Kamay, A Baden

**Affiliations:** 1 Department of Public Health, Faculty of Health Sciences, University of Niamey, Niger; 2 Health Sector Control Unit against STI / HIV / AIDS , Ministry of Public Health, Niger; 3 Niamey National Hospital, Niamey, Niger

## Introduction

Nosocomial infections recur periodically on the front of the stage and are nowadays recognized as major public health problems due to their frequency, severity and cost. Programs have been developed to slow the progression of nosocomial infections.

Despite all these efforts, nosocomial infections remain a persistent problem. In Africa, as in most developing countries, there are no national data on the prevalence of nosocomial infections.

## Objectives

To contribute to improving the quality of care and professional practices has an impact on the risk of infection.

## Methods

Prospective cross-sectional study made at the Centre Hospitalier Régional (CHR) Tahoua over a period of four months (June to September 2008) using a questionnaire and review of patient records to identify patients having contracted a nosocomial infection.

It involved patients of both sexes and all ages, and in some cases their carers and health workers identified in the various departments of Tahoua CHR located 600 km from Niamey.

## Results

Patients older than 56 years have contracted the largest number of nosocomial infections with a rate of 30.89% of cases. The departments of female Medicine and Surgery recorded the highest rates of nosocomial infections with respectively 26.7% and 27.74%.

More than nine out of ten patients with nosocomial infections (92.67%) did not have an invasive device. The average length of stay due to nosocomial infection is 4 days.

## Conclusion

Nosocomial infection is a constant concern in hospital practices of our country. Morbidity, mortality and cost caused by these infections justify the establishment of structures for monitoring, prevention and treatment.

## Competing interests

None declared

